# Assessment of inhalation flow patterns of soft mist inhaler co-prescribed with dry powder inhaler using inspiratory flow meter for multi inhalation devices

**DOI:** 10.1371/journal.pone.0193082

**Published:** 2018-02-20

**Authors:** Daiki Hira, Hiroyoshi Koide, Shigemi Nakamura, Toyoko Okada, Kazunori Ishizeki, Masafumi Yamaguchi, Setsuko Koshiyama, Tetsuya Oguma, Kayoko Ito, Saori Funayama, Yuko Komase, Shin-ya Morita, Kohshi Nishiguchi, Yasutaka Nakano, Tomohiro Terada

**Affiliations:** 1 Department of Pharmacy, Shiga University of Medical Science Hospital, Otsu, Shiga, Japan; 2 Department of Clinical Pharmacy, Faculty of Pharmaceutical Sciences, Kyoto Pharmaceutical University, Kyoto, Kyoto, Japan; 3 Hitachi Automotive Systems, Ltd., Isesaki, Gunma, Japan; 4 Department of Medicine, Division of Respiratory Medicine, Shiga University of Medical Science, Otsu, Shiga, Japan; 5 Kinki-Chozai Kadono Sanjo Pharmacy, Kyoto, Kyoto, Japan; 6 Oguma Family Clinic, Kusatsu, Shiga, Japan; 7 Oral Rehabilitation, Niigata University Medical and Dental Hospital, Niigata, Niigata, Japan; 8 Department of Respiratory Internal Medicine, St. Marianna University, School of Medicine, Yokohama-City Seibu Hospital, Yokohama, Kanagawa, Japan; University of Sydney, AUSTRALIA

## Abstract

The patients’ inhalation flow pattern is one of the significant determinants for clinical performance of inhalation therapy. However, the development of inhalation flow meters for various inhalation devices has been unable to keep up with the increasing number of newly launched inhalation devices. In the present study, we developed simple attachment orifices for the inhalation flow pattern monitoring system, which are suitable for all commercial inhalers, and investigated the efficacy of the system on the clinical inhalation instruction for patients co-prescribed dry powder inhaler (DPI) and soft mist inhaler (SMI). First, we constructed simple attachment orifices that were adjusted for 13 commercial inhalers, and examined the correlation between orifice and inhalation device. Second, the inhalation flow patterns (peak inspiratory flow rate, PIFR; inhalation duration time, DT) of patients prescribed a combination of DPI and SMI were monitored before and after inhalation instruction. The inhalation resistance of commercial inhalers are listed in the following order; Twincaps^®^ > Handihaler^®^ > Swinghaler^®^ = Clickhaler^®^ > Twisthaler^®^ > Turbuhaler^®^ > Jenuair^®^ > Diskus^®^ = Ellipta^®^ > Diskhaler^®^ > Breezhaler^®^ > Respimat^®^ = pMDI. The pressure drop via orifice was significantly correlated with that via the commercial inhaler. For the confirmation, all participants achieved the DPI criterion of PIFR. On the other hand, 4 participants (6 clinical visits) of 10 experimented participants could not achieve the essential criterion of DT (> 1.5 sec) for SMI, but all participants improved their duration time after inhalation instruction by pharmacists (P<0.05). In the present study, we successfully developed simple attachment orifice suitable for 13 commercial inhalation devices. These data suggested that our simple attachment orifices for the inhalation flow pattern monitoring system can detect patients with inadequate inhalation patterns via SMI.

## Introduction

Inhalation therapy is an established way for the treatment of asthma and chronic obstructive pulmonary disease (COPD). The efficacy of inhalation therapy is expressed by drug delivery to the treatment area, such as lungs and bronchus. The amount of drug delivered to the area is determined by the patient’s inhalation flow [1]. Therefore, the patients’ inhalation flow pattern is one of the significant determinants for clinical performance of inhalation therapy [2–4]. However, incorrect use of inhaler device by patient has been a major problem, which spoils the benefits of inhalation therapy [3]. Many studies for dry powder inhalers (DPI) have demonstrated that a high inhalation flow rate is required to disperse the micronized drug particle [5–7]. On the other hand, too high of an inhalation flow rate seems to decrease the pulmonary deposition rate of micronized drug particles, which aerolized by DPIs as well as nebulizer [5, 8]. In addition, the optimal inhalation flow rate is different for each inhalation device. In some commercial inhalers, the required flow rate is determined to release the drug particles from an inhaler [9]. As the measurement method of inhalation flow rate, we previously reported the inhalation flow pattern monitor system [5, 10, 11]. In clinical practice, In check^®^ and whistles for some devices are available [12]. However, development of inhalation flow meters for all inhalers has been unable to keep up with the increasing number of newly launched inhalation devices [9, 13, 14]. Therefore, development of a cyclopedic measurement system for inhalation flow patterns from commercial inhalers is clinically desired.

As a novel inhalation device, the soft mist inhaler (SMI) is a new type of propellant-free inhaler that generates a fine aerosol mist. This novel device has been shown to deliver a higher proportion of the emitted dose to the lung than a pressurized metered dose inhaler (pMDI) or DPI [15–18]. Furthermore, SMI is easier than pMDI to coordinate the actuation of the device with inhalation because the mist from SMI continues for 1.5 seconds [16]. On the other hand, in the case of SMIs, in contrast with DPIs, medical professionals should assess not only inhalation flow rate, but also comprehensive inhalation pattern including inhalation duration time (> 1.5 sec).

In the present study, we have developed simple attachment orifices for the inhalation pattern measurement system, which are suitable for all commercial inhalers, and investigated the efficacy of the clinical inhalation instruction for patients co-prescribed DPI and SMI using this system.

## Methods

### 1. Development of the attachment orifices suitable for different inhalers

**Fig 1:**
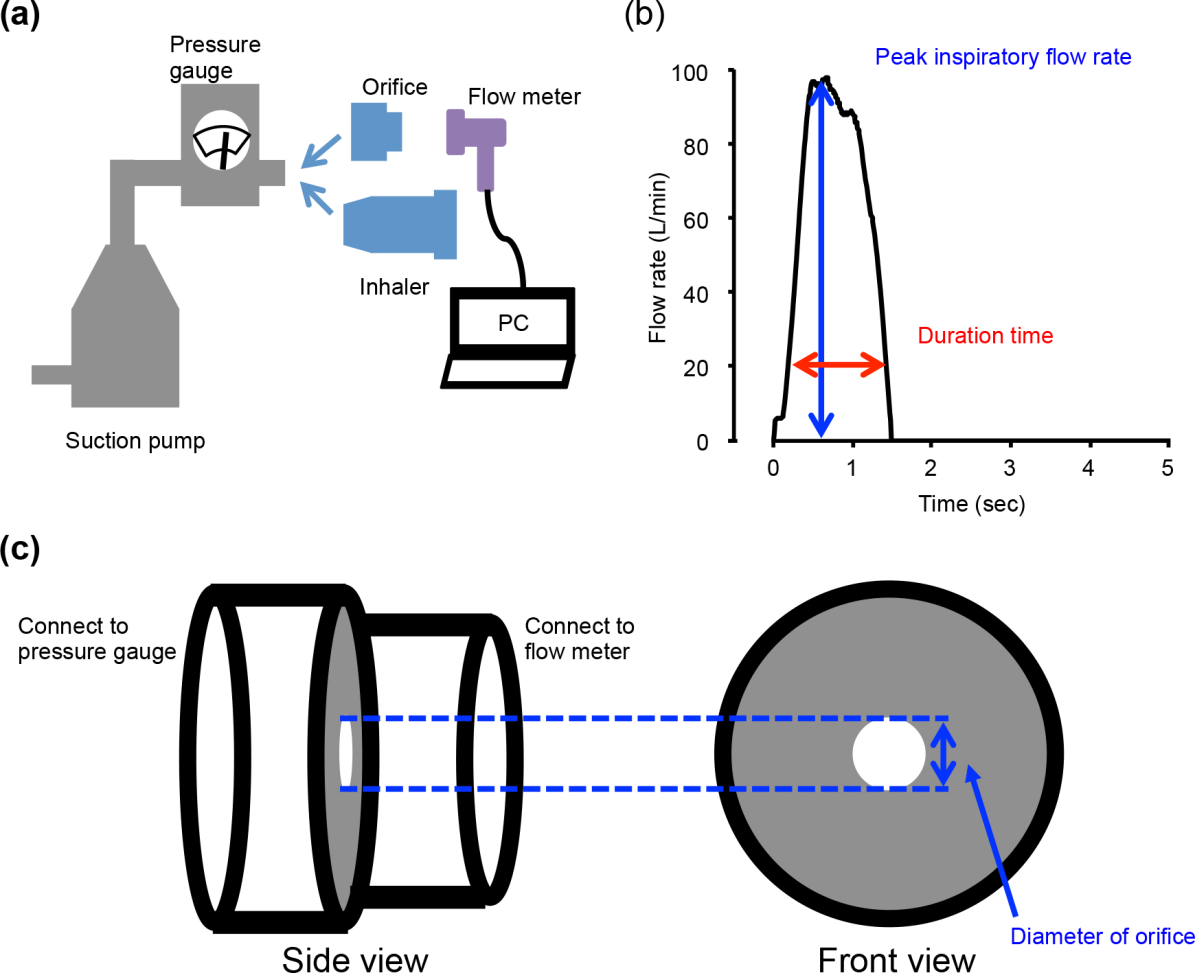
Schematic diagrams of inspiratory flow rate and the pressure drop monitoring system (a), typical inhalation flow pattern and parameters (b), and Schematic diagrams of attachment orifice (c).

The inspiratory flow recorder with a pressure gauge (Hitachi Automotive Systems, Ltd., Japan) reported in our previous report [11] was used to measure and visualize the inspiratory flow patterns and pressure drops via all commercial inhalers available in Japan. As shown in Fig 1, the inspiratory flow recorder with a pressure gauge, which consisted of a hot-wire flow meter, a pressure gauge, a power-supply box, and a personal computer. The hot-wire flow meter was applied for high time resolution (milli-second order) and low flow resistance. The pressure gauge was confirmed not to influence the measurements of inspiratory patterns.

**Fig 2:**
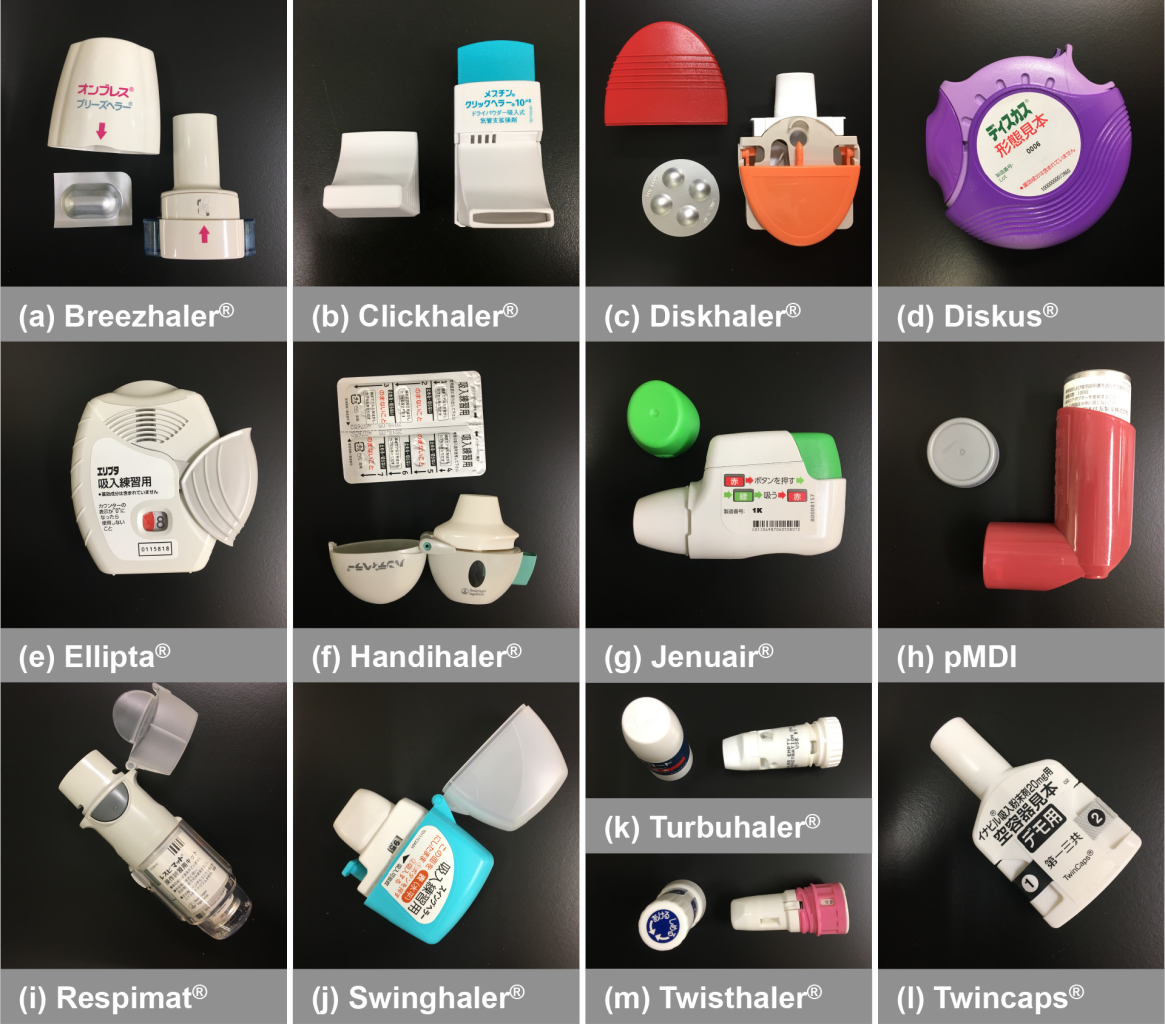
Inhalation devices commercially available in Japan. a, Breezhaler^®^ (Novartis Pharma AG, Switzerland.); b, Clickhaler^®^ (Otsuka Pharmaceutical Co., Ltd., Japan); c, Diskhaler^®^ (GlaxoSmithKline, UK); d, Diskus^®^ (GlaxoSmithKline, UK); e, Ellipta^®^ (GlaxoSmithKline, UK); f, Handihaler^®^ (Boehringer Ingelheim GmbH, Germany); g, Jenuair^®^ (KYORIN Pharmaceutical Co., Ltd., Japan); h, pMDI (3M, USA); i, Respimat^®^ (Boehringer Ingelheim GmbH, Germany); j, Swinghaler^®^ (Otsuka Pharmaceutical Co., Ltd., Japan); k, Turbuhaler^®^ (AstraZeneca plc, UK); l, Twincaps^®^ (Daiichi Sankyo co., Ltd., Japan); and m, Twisthaler^®^ (Merck and Co., USA).

Thirteen inhalation devices commercially available in Japan (Fig 2) were used for the present study: Breezhaler^®^ (Novartis Pharma AG, Switzerland.), Clickhaler^®^ (Otsuka Pharmaceutical Co., Ltd., Japan), Diskhaler^®^ (GlaxoSmithKline, UK), Diskus^®^ (GlaxoSmithKline, UK), Ellipta^®^ (GlaxoSmithKline, UK), Handihaler^®^ (Boehringer Ingelheim GmbH, Germany), Jenuair^®^ (KYORIN Pharmaceutical Co., Ltd., Japan), pMDI (3M, USA), Respimat^®^ (Boehringer Ingelheim GmbH, Germany), Swinghaler^®^ (Otsuka Pharmaceutical Co., Ltd., Japan), Turbuhaler^®^ (AstraZeneca plc, UK), Twincaps^®^ (Daiichi Sankyo co., Ltd., Japan), and Twisthaler^®^ (Merck and Co., USA). Customized low volume air sampler, Andersen type, model AN-200 (Shibata Scientific Technology Ltd., Japan) or System for Testing the Dose Uniformity of DPIs (Copley scientific Ltd., U.K.) were connected to the inhalation flow recorder via inhalation devices or corresponding attachment orifices, which were adjusted to each device by regulation of diameter of orifice (Fig 1C). Here, the orifice is an inhalation flow restriction plate attached on an inhalation flow meter, and is able to simulate the inhalation resistance of each inhalation device by adjusting its diameter and shape. The diameter and shape of orifices for each device were adjusted based on the pressure drop of each device at a 30 L/min inspiratory flow rate. Consecutively, the relationship between orifice and inhalation device pressure drop was confirmed with a 10 to 90 L/min inspiratory flow rate. The orifices were constructed by referencing the shape of In-Check^®^ (Clement Clark Ltd, UK), which is a commercially available inhalation flow rate meter. The In-Check^®^ orifices were also applied for the measurement of Diskus, Diskhaler, Handihaler, and Turbuhaler, which are able to be measured by In-check^®^ orifices [19]. Pearson correlation analysis was used to examine the correlation between orifice and inhalation device. Bland Altman analysis was used to assess differences and biases between orifice and inhalation device. The bias was calculated by mean difference between orifice and inhalation device. The limit of agreement was calculated by 1.96 × standard deviation of difference between orifice and inhalation device. The measurement was performed in triplicate. The device specific resistance (R_D_) of each device was calculated by the least squares fitting method with the following equation:

$${\Delta P}^{0.5}=R_{D}\times Q$$ Eq 1

where **Δ**P is pressure drop via inhalation device and Q is the volumetric flow rate [20].

### 2. Impact of inhalation flow pattern assessment on inhalation instruction

The present study was conducted in Shiga University of Medical Science Hospital from February to July 2016. Inclusion criteria were the adult patients who were prescribed a combination of DPI and SMI. Exclusion criteria were the patients who refused the informed consent, or age younger than 20 years. A total of 10 participants were measured their inhalation flow patterns via their prescribed devices at their regular clinical visits (1–3 clinical visits in a participant). Peak inhalation flow rate (PIFR) and inhalation duration time (DT) were assessed as parameters of inhalation pattern (Fig 1B). The essential criterion of optimal inhalation pattern for DPI and SMI were (1) higher than the lower limit of PIFR for each device in order to disperse dry powder from the inhalation device and (2) a DT longer than 1.5 sec for soft mist spraying time [15], respectively. The lower limits of PIFR for each device are listed in Table 1 [21–29].

**Table 1:** Lower limit of peak inhalation flow rates for commercial dry powder inhalers.

Device	Optimal peak inhalation flow rate (PIFR) for powder dispersion	Reference
Respimat^®^, pMDI	Lower is better	[8]
Clickhaler^®^	> 20 L/min	[22]
Handihaler^®^		[21]
Swinghaler^®^		-
Twincaps^®^		-
Diskus^®^	> 30 L/min	[23]
Ellipta^®^		[24]
Turbuhaler^®^		[25]
Twisthaler^®^		[26]
Jenuair^®^	> 45 L/min	[27]
Breezhaler^®^	> 50 L/min	[28]
Diskhaler^®^	> 60 L/min	[29]

The clinical protocol for instruction of inhalation pattern is as follows. In the case that the participant could not achieve the criterion, participants were received the instruction for inhalation by pharmacists, and measured their inhalation pattern again. The achievement rate of the criterion between pre- and post- instruction were analysed with Chi square test. In addition, the difference of inhalation pattern parameters between pre- and post- instruction were compared and analysed with the paired *t*-test. A difference was considered significant at P< 0.05. This study was performed in accordance with the Declaration of Helsinki. This human study was approved by the Ethics Boards of Shiga University of Medical Science—approval: 27-14-1. All participants provided written informed consent to participate in this study.

## Results

### 1. Development of the attachment orifices suitable for different inhalers

**Fig 3:**
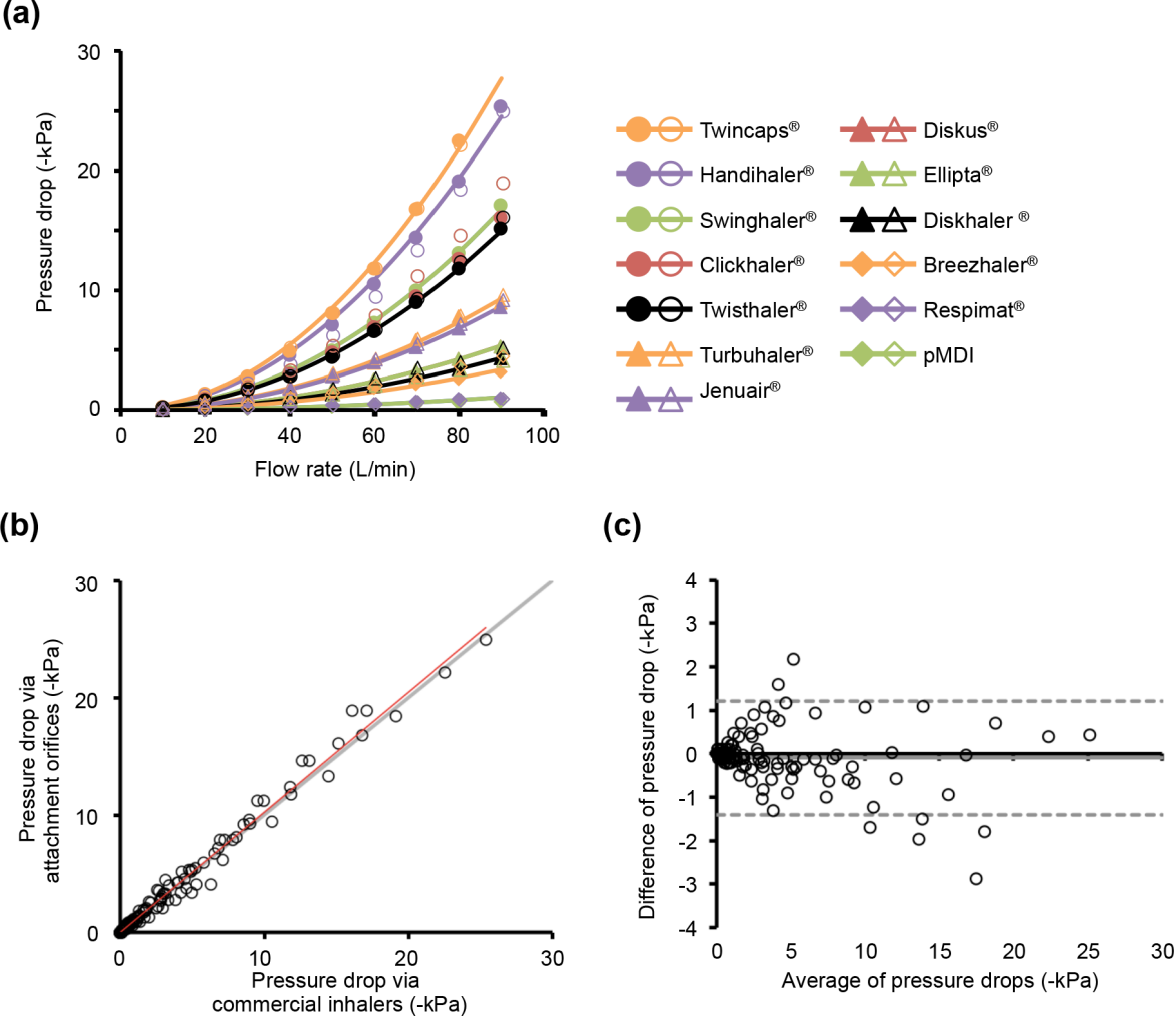
Relationships between inspiratory flow rate and pressure drop of commercial inhalers (filled points) and simple attachment orifices (open points) (a). Relationships of pressure drops between commercial inhalers and orifices (b). Gray and red lines represent y = x and approximated line (y = 1.024 x + 0.003, R^2^ = 0.9851), respectively. Bland-Altman plot for relationship of pressure drops between commercial inhalers and orifices (c). Gray solid and dotted lines represent bias and 95% limit of agreement interval of difference of pressure drop between commercial inhalers and orifices (-0.10, -1.38 to 1.18), respectively.

Fig 3A shows the relationships between inhalation flow rate and pressure drop of each inhalation device. A high pressure drop at the same inhalation flow rate indicates high inhalation resistance. The inhalation resistance of commercial inhalers are listed in following order; Twincaps^®^ > Handihaler^®^ > Swinghaler^®^ = Clickhaler^®^ > Twisthaler^®^ > Turbuhaler^®^ > Jenuair^®^ > Diskus^®^ = Ellipta^®^ > Diskhaler^®^ > Breezhaler^®^ > Respimat^®^ = pMDI. In the case of the orifice constructed based on each commercial inhaler, the relationships between inhalation flow rate and pressure drop were similar with commercial inhalers, and fit well with Eq 1 (Fig 3A). The R_D_ values calculated by Eq 1 were negatively correlated with orifice diameter (Table 2). In addition, pressure drop via the orifice was significantly correlated with that via the commercial inhaler (R^2^ = 0.985, P < 0.05, Fig 3B). The Bland-Altman plot showed no additional bias between the orifice and the commercial inhaler (bias = -0.10, 95% limit of agreement interval = -1.38 to 1.18, Fig 3C).

**Table 2:** Device-specific resistance and orifice diameters.

Device	R_D_ (Pa*min^2^/L^2^)	Diameter of orifice (mm)
Respimat^®^, pMDI	0.13	10
Breezhaler^®^	0.42	5.8
Diskhaler^®^	0.54	4.83
Diskus^®^, Ellipta^®^	0.66	5.38
Jenuair^®^	1.07	4.2
Turbuhaler^®^	1.15	4.03
Twisthaler^®^	1.85	3.6
Swinghaler^®^, Clickhaler^®^	2.07	3.5
Handihaler^®^	3.03	3.33
Twincaps^®^	3.42	3.6

R_D_: device specific resistance

### 2. Impact of inhalation flow pattern assessment on inhalation instruction

**Fig 4:**
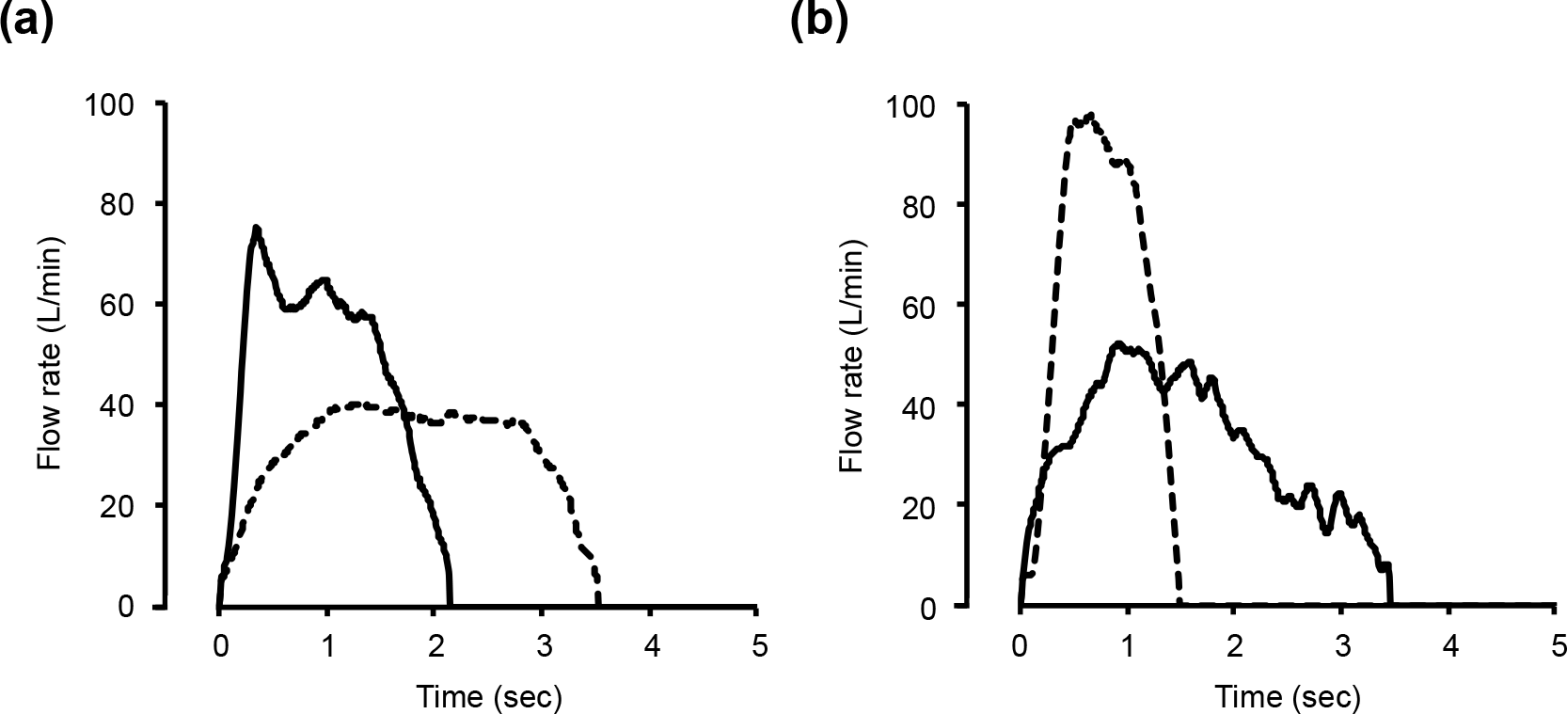
**Typical inspiratory flow profiles of a dry powder inhaler (Diskus**^**®**^**, a) and soft mist inhaler (b).** Dashed and solid lines represent before and after inhalation instruction, respectively.

**Fig 5:**
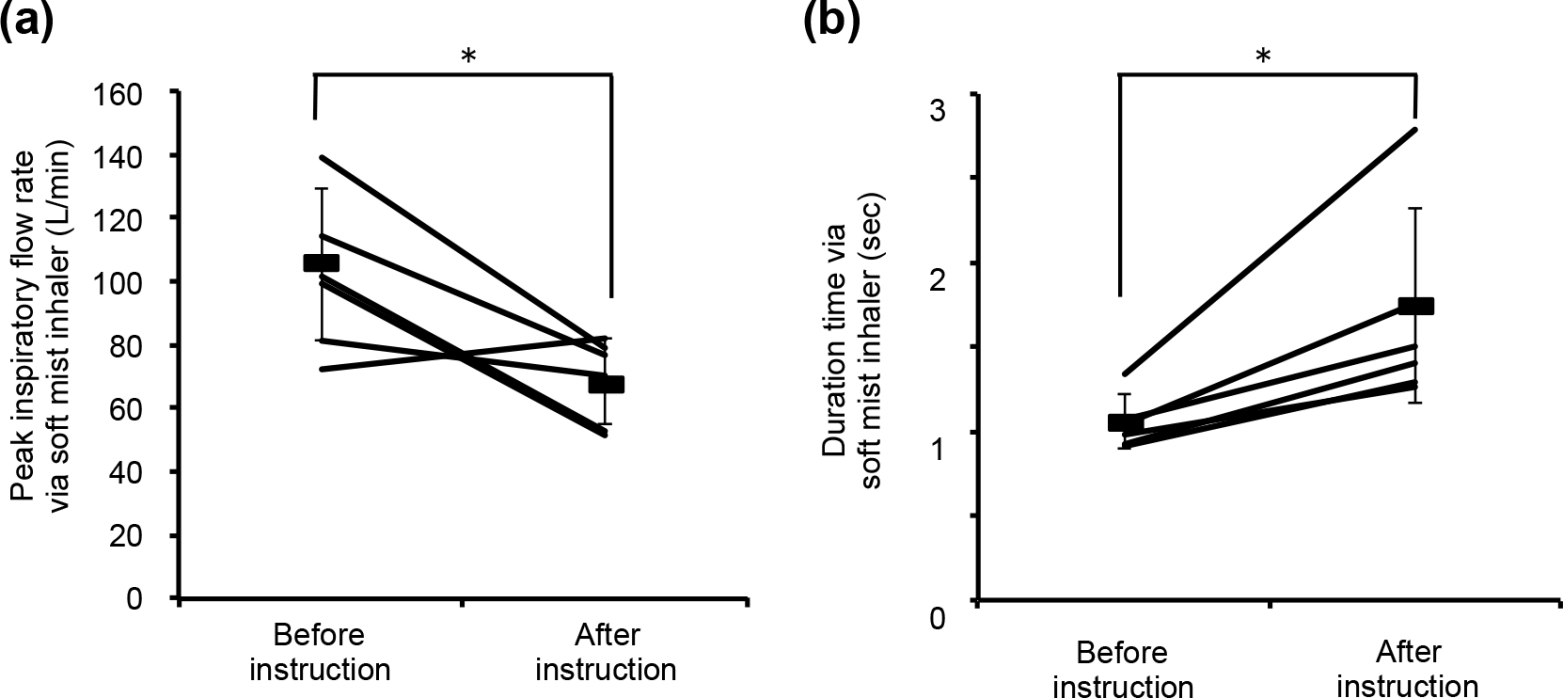
**Participants’ peak inspiratory flow rate (a) and duration time (b) via soft mist inhaler, before/after inhalation instruction.** Black bars represent mean ± SD. Asterisk (*) indicates statistical significance (P < 0.05).

**Fig 6:**
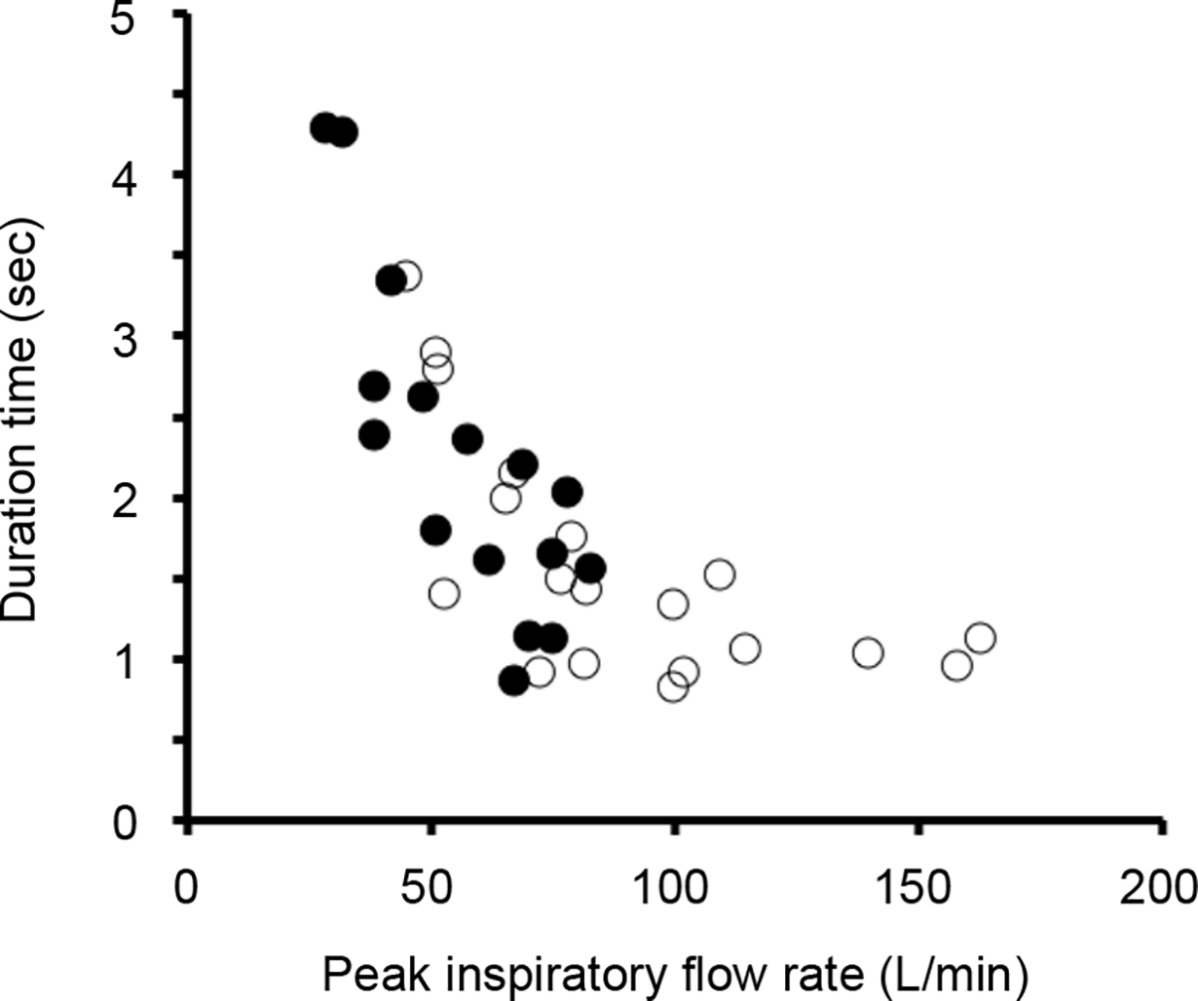
Relationship between peak inspiratory flow rate and duration time. Data including both of before and after inhalation instruction. Filled and open circles represent dry powder inhaler and soft mist inhaler, respectively.

A total of 10 participants prescribed both DPI and SMI gave written informed consent and their inhalation flow patterns were measured. The participants’ characteristics were summarized in Table 3. Among them, all participants achieved the DPI criterion of PIFR. On the other hand, 4 participants (6 clinical visits) could not achieve the essential criterion of duration time for SMI. After inhalation instruction, their inhalation pattern was measured again, and the difference between pre- and post- instruction was analysed. Fig 4 shows the typical inhalation profiles of DPI and SMI. A typical error observed with participants prescribed both DPI and SMI is the rapid inhalation with DPI causing a shorter inhalation duration time for SMI due to confused usage of DPI and SMI. In the case in Fig 4, the participant achieved the DPI (Diskus^®^) criterion (PIFR > 30 L/min) at pre- and post- instruction; however, the SMI criterion (DT > 1.5 sec) was not achieved at pre-instruction. After inhalation instruction by pharmacists, the achievement rate of SMI criteria after the instruction (3/6, 50%) is significantly higher than that of pre-instruction (0/6, 0%, P < 0.05). As shown in Fig 5, DT of all participants was significantly prolonged after inhalation instruction, but PIFR of SMI decreased (P < 0.05). The relationship between PIFR and DT (Fig 6) indicated that lower PIFR caused a longer DT with both SMI and DPIs.

**Table 3:** Participant characteristics.

	Median (Range)
Age (years)	80 (25–88)
	**No. of participants**
Gender (Female / Male)	1/9
Types of dry powder inhaler (Turbuhaler ® / Diskus ® / Breezhaler ®)	4/4/2
Types of soft mist inhaler (Respimat ®)	10

## Discussion

### 1. Development of the attachment orifices suitable for different inhalers

Inhalation instruction plays an important role in inhalation therapy for asthma and COPD because therapeutic efficacy of inhalation medicine is influenced by the patient’s inhalation technique [3, 30–33]. Therefore, it is an urgent issue to develop inhalation instruction tools for a large variety of inhalation devices [12]. In the present study, we successfully developed simple attachment orifices for the inhalation flow pattern monitoring system, which are suitable for 13 commercially available inhalers. Furthermore, from clinical application of the monitoring system, we demonstrated that the monitoring system is useful for detection and instruction of inadequate inhalation flow patterns via SMI in participants prescribed both DPI and SMI. As shown in Fig 3, the orifices constructed in the present study had a markedly high correlation and less biases with commercial inhalers. Therefore the orifices can be used in clinical practice with high accuracy. There are wide differences in inhalation resistance of DPIs because DPIs have a complicated structure in order to micronize the drug particle by the patient’s inhalation flow [11]. However, our orifice also mimicked the inhalation resistance from simply structured orifices to complicatedly structured devices.

### 2. Impact of inhalation flow pattern assessment on inhalation instruction

In clinical practice, there are many reports of inadequate usage of DPIs and MDIs [30, 34], but there is less information for SMI. For DPIs, a higher PIFR is desired to micronize drug particles [5]. On the other hand, for MDIs and SMI, a lower PIFR is desired to prevent the deposition to the oropharyngeal area by inertial force [8]. In addition, for SMI, a longer DT is desired to recover the 1.5-second sustained mist [15]. Due to the large difference between DPI and SMI as described above, co-prescription of DPI and SMI causes serious confusion in inhalation therapy. In the present study, some participants made inadequate inhalation patterns with SMI due to confusion with DPIs. For such participants, their inhalation patterns were significantly improved after inhalation instruction with the inhalation flow pattern monitoring system, which is able to visualize their inhalation flow pattern in real time. Although the orifices in the present study were constructed by referencing the shape of In-Check^®^, a commercially available PIFR meter [19], In-Check^®^ cannot quantify DT via SMI and PIFR via novel inhalation devices, such as Breezhaler^®^ and Jenuair^®^. Therefore, the inhalation flow pattern monitoring system has the advantage in inhalation instruction. On the other hand, since our orifice can be used as the attachments of In-Check^®^, our orifices connected to In-Check^®^ can be used for PIFR assessment of novel inhalation devices, but not for DT via SMI. It has been reported that lower inhalation resistance causes a higher PIFR at the same pressure drop [11, 35]. Therefore, SMI has a low inhalation resistance and results in a high PIFR. As a lower PIFR made a longer DT, as shown in Fig 5C, conscious inhalation instruction for slower PIFR and longer DT is required in the case of SMI. This instruction has clinical and pharmaceutical impact because a slower PIFR decreases the inertial force on inhaled drug particles and mists, and makes the drugs spread throughout the whole lung [5, 8]. Recently, the TRILOGY study demonstrated the effectiveness of triple therapy combining an inhaled corticosteroid, a long-acting β_2_ agonist, and a long-acting muscarinic antagonist via single inhaler [36]. Since the triple combination therapy became one of a therapeutic option, patients who prescribed multiple inhalation therapies may increase. However, there is no commercially available inhaler contains these three drugs in a device. Therefore, patients who prescribed multiple inhalation devices may also increase. In clinical practice, clinicians should pay attention to the patients’ confusion with complicated inhalation therapy with multiple inhalation devices.

In conclusion, we constructed simple attachment orifices for the inhalation flow pattern monitoring system suitable for 13 commercial inhalation devices. The small number of participants and no clinical influence of short DT via SMI are limitations of the present clinical study. Further study will be required to evaluate the clinical influence of short DT via SMI in a prospective trial. Although this study was preliminary, the novel system successfully detected patients with inadequate inhalation patterns (short DT) via SMI, and confirmed prolongation of their DT via SMI after inhalation instruction. The real time inhalation flow pattern monitoring system with simple attachment orifices is a promising system to achieve adequate inhalation with DPI and SMI.
